# Carfilzomib induced cardiotoxicity in a multiple myeloma patient

**DOI:** 10.1186/s40959-020-00074-8

**Published:** 2020-09-07

**Authors:** Arnold Méndez-Toro, Cándida Díaz-Brochero, Estivalis Acosta-Gutiérrez

**Affiliations:** 1 Cardiology Unit, Universidad Nacional de Colombia, Hospital Universitario Nacional de Colombia, Bogotá, Colombia; 2 Department of Internal Medicine, Universidad Nacional de Colombia, Hospital Universitario Nacional de Colombia, Bogotá, Colombia

**Keywords:** Proteasome inhibitor, Multiple myeloma, Carfilzomib, Heart disease, Heart failure, Cardiotoxicity

## Abstract

Proteasome inhibitors such as carfilzomib are indicated in multiple myeloma patients showing relapse and/or refractoriness of clonal activity. However, this therapy has been associated with a significant incidence of cardiotoxicity, especially in patients with known cardiovascular risk factors. Here we report a case of a 60-year-old woman with multiple myeloma, who developed severe congestive heart failure with positive myocardial injury biomarkers together with impaired LVEF and GLS, after treatment with carfilzomib. Therefore, chemotherapeutic drug was discontinued and neurohormonal blocking and diuretic therapy was started resulting in amelioration of symptoms, without changes in LVEF but with significant GLS improvement. Although high-grade cardiotoxicity is relatively rare in patients with non previous cardiac risk factors, it was a predictable side effect of carfilzomib use. Recognition of this syndrome is critical to instauration of appropriate therapy and prevention of morbimortality.

## Background

Multiple myeloma (MM) is a clonal plasma cell pathology that represents approximately 10% of the malignant hematological disorders. Average survival is approximately 5 to 7 years, with variations according to individual’s characteristics, tumor stage, cytogenetic alterations, and treatment response [1]. The phases of cancer treatment include initial therapy with immunomodulators, protease inhibitors, and dexamethasone. Subsequently, if the patient is eligible, autologous stem cell transplant (ASCT) is performed. A maintenance phase follows, and its duration varies according to the identified cytogenetic profile and individual risk factors. Finally, the last phase consists of treating patients with refractoriness or relapse despite established management. In the latter case, triple therapy with immunomodulators, dexamethasone, and proteasome inhibitors (PI) such as carfilzomib is indicated. The function of carfilzomib is to irreversibly inhibit the protease activity of 20S proteasome, −which is responsible for inter-cellular protein degradation through the ubiquitin-proteasome–, and to disrupt cellular signaling pathways, leading the cell to apoptosis [2].

One of the most relevant adverse events of carfilzomib is its cardiotoxicity, which covers a broad range of clinical signs and symptoms classified into five categories according to its severity: 1: mild, 2: moderate, 3: severe, 4: life threatening or disabling, and 5: fatal. The last three categories described above correspond to high-grade cardiovascular adverse events (CVAE) [3, 4].

Below, we discuss a case of a MM patient with tumor relapse, who developed severe congestive heart failure after treatment with carfilzomib. Therefore, the medication was discontinued, and neurohormonal blocking therapy was initiated showing subsequent clinical and echocardiographic improvement.

A 60-year-old female with MM diagnosis since 2016, presented to the hospital on December, 2019. She had chronic anemia (Hb 8 g/dL), stage 3A- A3 chronic kidney disease (GFR 52 ml/min/1.73 m2), and non-nephrotic proteinuria (1.8 g in urine collection over 24 h). Regarding the oncological treatment, on April, 2016, she underwent eight CyBorD cycles (Cyclophosphamide, Bortezomib, Dexamethasone). Later, on March 2018, an ASCT was performed. Then, maintenance therapy with lenalidomide and dexamethasone was started. Nevertheless, due to disease progression, a KRD chemotherapy plan was prescribed (Leflunomide, Carfilzomib, Dexamethasone). A transthoracic echocardiogram prior to the initiation of the therapy evidenced a preserved ventricular systolic function LVEF 58% and GLS of − 17%.

After 5 cycles of treatment, the patient experienced a rapid decline in her functional status –NYHA class from I to II – in 2 weeks, paroxysmal nocturnal dyspnea and orthopnea, so she was admitted to the hospital. Based on international recommendations related to monitoring and treatment of CVAE in patients with MM treated with carfilzomib [4], diagnostic studies were indicated. These included serum myocardial injury biomarkers: NT-proBNP of 17,570 pg/mL (cut-off point ≥900 pg/mL in patients between 50 and 75 years-old) and troponin I of 0.006 (cut-off point 0–0.017 pg/ml). A transthoracic echocardiogram showed left ventricular concentric hypertrophy, 182 g/m2 of ventricular mass, diffuse hypokinesia, LVEF of 45% and GLS of − 11.7%. Based on these findings, congestive severe heart failure secondary to carfilzomib toxicity was considered, classified as a high-grade CVAE. It was decided to discontinue the therapy. The following prescription was initiated: enalapril 5 mg twice a day, carvedilol 25 mg twice a day, spironolactone 25 mg daily, and intravenous furosemide 10 mg four times a day.

Also, bone marrow studies were performed including flow cytometry, which did not show plasmocytes. Monoclonal component showed protein immunofixation in urine in low quantities, suggesting good partial response to previous treatment. Nevertheless, since the patient had previously shown disease progression with a combination of lenalidomide and dexamethasone, the hematology department decided to add daratumumab (human IgGk monoclonal antibody targeted against CD38) as third line of management.

**Fig. 1:**
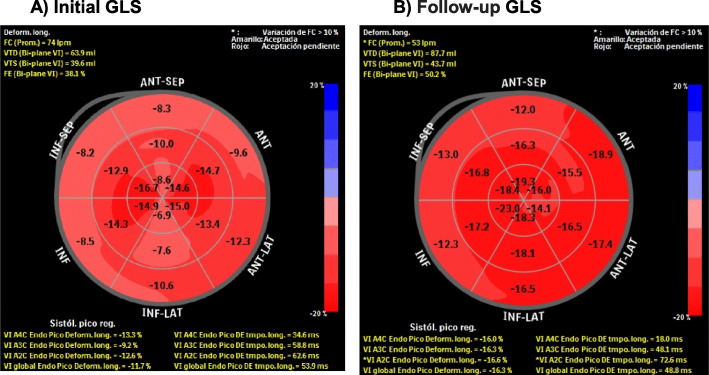
**a** GLS reduction after 4 months of carfilzomib treatment. **b** GLS improving after cardiotoxicity treatment and 2 months after carfilzomib was discontinued

Forty-five days after discontinuing carfilzomib, during outpatient monitoring at cardiology unit, she reported clinical improvement of dyspnea and orthopnea. A follow-up echocardiogram did not show changes in LVEF but demonstrated significant GLS improvement, −current value of − 16.3 and prior value of − 11.7 – (Fig. 1).

## Discussion

Patients with MM have a greater probability of developing cardiovascular events than general population independent of the established cancer treatment [5]. Among the known risks factors there are those related to the patient (age and other classic cardiovascular risk factors), those specifically related to the disease (chronic anemia, kidney failure, hyperviscosity, amyloidosis, proteinuria), and those related to oncological treatment itself such as the receptors of ASCT or specific chemotherapeutic cardiotoxicity [6–8].

Survival rates of MM patients with relapse or refractoriness to more than one line of treatment have improved in a significant manner with the development of PI, including carfilzomib. Nevertheless, this group of medications have been associated with an increase incidence of CVAE, which is dosage dependent [2]. Recently, a meta-analysis [9] reported that from 2594 patients with MM treated with carfilzomib, 18.1% had CVAE, 8.2% of them being high-grade CVAE. Heart failure (4.1%) and high blood pressure (12.2%) were the most common cardiovascular events, while arrhythmia (2.4%) and ischemic events (1.8%) occurred less frequently. These findings represent a larger incidence of cardiotoxicity compared to other PI such as bortezomib, which shows a high-grade CVAE incidence of 2.3% [2, 3]. Possibly it can be explained by a more potent effect of bortezomib, which also inhibit the β unit of proteasome 20S irreversibly [10]. In this case, the patient had previously been treated with bortezomib, with no related CVAE demonstrated. It was not until the start of carfilzomib treatment that the patient developed severe heart failure.

Underlying mechanisms related to cardiovascular complications associated with carfilzomib have not been completely established. Murine models have shown that administration of sub-micromolar concentrations of carfilzomib generates inhibition of proteasome activity similar to chymotrypsin, with subsequent damage of cardiac myocytes and apoptosis induction [2, 11]. High doses of PI have also been reported to negatively regulate nitric oxide synthase function, resulting in endothelial vascular dysfunction and increased cardiovascular risk [12].

Prevention of cardiotoxicity induced by carfilzomib, as well as other IP, is based on the management of known modifiable risk factors, reduction of the administered dose or temporary medication interruption in cases where high-grade CVAE have been demonstrated. Additionally, studies in which anthracycline cardiotoxicity has been analyzed, the use of medications such as ACEI or ARBs and beta blockers has shown to be effective in limiting the development of interstitial fibrosis, intracellular oxidative stress reduction, and intracellular calcium cycle metabolism enhancement, which presumably could prevent the development of ventricular dysfunction [4, 13]. Based on these findings, international experts [4] have suggested the administration of these medications in those patients with carfilzomib cardiotoxicity.

A retrospective cohort study showed that 16.3% MM patients treated with carfilzomib decreased their LVEF during treatment. Nevertheless, after interruption of carfilzomib and initiation of beta-blockers, angiotensin converting enzyme inhibitors and diuretics, all patients improved their systolic function in two months average [14]. In our case, after the interruption of carfilzomib and start of the neurohormonal blocking therapy, the patient showed improvement of dyspnea, as well as GLS value, with preserved LVEF. These data suggest that optimization of heart failure management and interruption of medication in high-grade CVAE have shown a positive effect in the improvement of symptoms and echocardiographic parameters.

Regarding the possibility of restarting the treatment with PI and when to do it, there is not conclusive evidence. Comparing the cardiovascular mortality rate in patients with MM (less than 10%) versus the mortality generated by cancer itself and its associated complications (overall 5-year survival rate of 54%), it can be challenging balancing the potential heart dysfunction caused by this chemotherapeutic agent with its positive effects in the treatment of MM patients [15, 16]. For this reason it is extremely important to offer interdisciplinary management with the cardio-oncology team, explore the patient preferences and discuss which treatment options best fits the patient needs [17].

The use of carfilzomib as a chemotherapeutic agent in the management of refractory MM have improved the pronostic of these patients at the expense of the development of potential cardiotoxicity. This case illustrates the behavior of carfilzomib induced CVAE and the expected response to the appropriate pharmacological management. Diagnosis of cardiotoxicity is based on symptomatology and investigational studies where diagnostic images play a crucial role for its follow-up and prognosis, as do the myocardial injury serum markers. Lacking appropriate management, high-grade CVAE can increase morbidity and mortality in the patients as well as costs to the health system.

## Data Availability

All data generated and analyzed during this study are included in this published article.

## Abbreviations

MM: Multiple Myeloma
LVEF: Left Ventricular Ejection Fraction
GLS: Global Longitudinal Strain
CVAE: Cardiovascular Adverse Events
ASCT: autologous stem cell transplant
CyBorD: Cyclophosphamide, Bortezomib, Dexamethasone
KRD: Leflunomide, Carfilzomib, Dexamethasone
NYHA: New York Heart Association’s functional classification for heart failure
PI: Proteasome Inhibitors
NT-PROBNP: N-terminal Pro Brain Natriuretic Peptide
ACEI: Angiotensin Converting Enzyme Inhibitors
ARB: Angiotensin Receptor Blocker
